# Long-lasting response to electrochemotherapy in melanoma patients with cutaneous metastasis

**DOI:** 10.1186/1471-2407-13-564

**Published:** 2013-12-01

**Authors:** Corrado Caracò, Nicola Mozzillo, Ugo Marone, Ester Simeone, Lucia Benedetto, Gianluca Di Monta, Maria Luisa Di Cecilia, Gerardo Botti, Paolo Antonio Ascierto

**Affiliations:** 1 Unit of Surgery “Melanoma - Soft Tissues – Head & Neck – Skin Cancers”, Istituto Nazionale per lo Studio e la cura dei tumori “Fondazione G.Pascale” IRCCS, Naples, Italy; 2 Unit of Medical Oncology, Istituto Nazionale per lo Studio e la cura dei tumori “Fondazione G.Pascale” IRCCS, Naples, Italy; 3 Unit of Pathology, Istituto Nazionale per lo Studio e la cura dei tumori “Fondazione G.Pascale” IRCCS, Naples, Italy

**Keywords:** Electrochemotherapy, Melanoma, Cutaneous metastases

## Abstract

**Background:**

Treatment of early and multiple cutaneous unresectable recurrences is a major therapeutic problem with around 80% of patients relapsing within 5 years. For lesions refractory to elective treatments, electrochemotherapy (ECT) involving electroporation combined with antineoplastic drug treatment appears to be a new potential option. This study was undertaken to analyze the short- and long-term responses of lesions treated with ECT with intravenous injection of bleomycin in melanoma patients with in-transit disease or distant cutaneous metastases.

**Methods:**

Between June 2007 and September 2012, 60 patients with relapsed and refractory cutaneous melanoma metastases or in-transit disease underwent 100 courses of ECT with intravenous injection of bleomycin. Response to treatment was evaluated three months after ECT. A long-lasting response was defined as no cutaneous or in-transit relapse after a minimum of six months.

**Results:**

Three months after ECT, a complete response was observed in 29 patients (48.4%), a partial response in 23 patients (38.3%) and no change or progressive disease in 8 patients (13.3%). The objective response rate of all treated lesions was 86.6%. Thirteen patients (44.8% of complete responders) experienced a long-lasting response after one ECT session and were disease-free after a mean duration of follow-up of 27.5 months.

**Conclusions:**

The favorable outcome obtained in the present study demonstrates that ECT is a reliable, and effective procedure that provides long-term benefit in terms of curative and palliative treatment for unresectable cutaneous lesions without adversely impacting the quality of life of patients.

## Background

Treatment of early and multiple cutaneous unresectable recurrences is a major therapeutic problem with around 80% of patients relapsing within 5 years [1]. In-transit metastases are cutaneous metastases that occur between a primary tumor and its regional lymphatic basin, with an incidence that varies from <5% in patients without nodal disease to 20% in cases with lymph node metastases [2]. The surgical excision of isolated lesions represents the standard treatment, while isolated limb perfusion may be adopted for multiple lesions involving the entire extremity.

For lesions refractory to elective treatments, electroporation appears to be a new potential therapeutic option. It is combined with intravenous administration of antineoplastic drugs such as bleomycin, and is referred to as electrochemotherapy (ECT). This study was undertaken to analyze the short- and long-term responses of melanoma patients with distant cutaneous metastases or in-transit disease undergoing ECT with intravenous injection of bleomycin. This is the first report to address the long-term response of patients with melanoma undergoing ECT.

## Methods

Between June 2007 and September 2012, 60 patients with relapsed and refractory cutaneous melanoma metastases or in-transit disease underwent one to five courses of ECT using the Cliniporator™ pulse generator with intravenous injection of bleomycin according to the European Standard Operating Procedures of Electrochemotherapy (ESOPE) guidelines [3].

All treatments were performed using the Cliniporator™ device (IGEA Ltd., Modena, Italy) with two different types of electrodes: type II for treatment of lesions in the head and neck area and type III for all other sites. Electric pulses were delivered from 8 until 28 minutes after intravenous injection of Bleomycin according to the ESOPE standardized criteria. The required dose of bleomycin was 15000 IU/m^2^; six (10%) patients had the dose reduced because of decreased renal function or cardiac disease. ECT was performed under general or loco-regional anesthesia, dependent on the area being treated. ECT treatment was performed after the approval of an appropriate ethics committee (IEC of National Cancer Institute of Naples, reference number 273/10) in compliance with Helsinki Declaration, following internationally recognized guidelines. All patients gave written informed consent before each treatment.

Before ECT, the most significant lesions were measured and photographed for each patient. Response to treatment was evaluated three months after ECT in accordance with World Health Organization (WHO) guidelines and defined as progressive disease (PD) for lesions increased in tumor size >25%, no change (NC) for lesions increased in tumor size <25% or decreased <50%, partial response (PR) for lesions decreased in tumor size >50% and complete responses (CR) for lesions that had disappeared. Patients were assessed every 4 weeks for six months and thereafter every three months. In the course of follow-up, the appearance of lesions in untreated areas was considered to be new disease rather than a relapse of the previously treated lesion. A further ECT session was proposed at least 6–8 weeks after the previous treatment in patients with lesions showing a PR. A long-lasting response was defined as no cutaneous or in-transit relapse after a minimum 6 months.

## Results

Clinical and pathological characteristics of patients are summarised in Table 1. All patients had recurrent cutaneous disease after one or more previous radical surgical treatments. Twenty-one patients had cutaneous or in-transit disease of the trunk, 35 had in-transit disease of an inferior limb and four patients had cutaneous disease in the head and neck area. No patient had previously received isolated limb perfusion or bleomycin.

**Table 1:** Patient and tumor characteristics

**Characteristic**	**Value**
Age, median (range), years	62 (27–89)
Male/female, *n*	34/26
Breslow thickness, median (range), mm	5.0 (0.8-20.0)
***Site of metastases, n***	
Trunk	21
Limbs	35
Head & neck	4
***Type of metastases, n***	
In-transit	35
Local recurrence	4
Distant cutaneous	21

A total of 100 courses of ECT were performed in the 60 patients; 34 patients had a single ECT session and 26 patients underwent ≥2 sessions of ECT, including one patient who had five sessions (Table 2). The mean rate of electrode application per patient was 73 and ranged from 8 to 187. The median duration of follow-up was 27.5 months (range 6–67 months). Treatment was well tolerated with the most frequent side effects being non-significant (i.e. mild) pain in 22 patients (36.6%) and myalgia in 8 patients (13.3%). No systemic side effects were observed. Necrosis of the treated lesions occurred in 18 patients (30.0%), all of whom had received multiple sessions of ECT. Tattoo of needle electrodes remained visible in the treated areas for about three months.

**Table 2:** Number of session of electrochemotherapy (ECT)

**Number of ECTs**	**Patients, *n***
1	60
2	25
3	8
4	6
5	1

**Figure 1:**
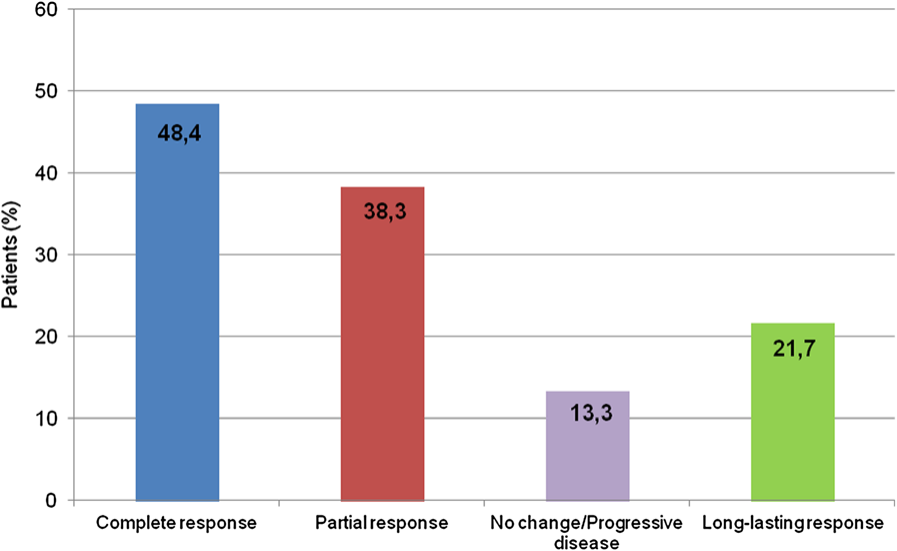
Treatment response.

Three months after the ECT session, 23 patients (38.3%) had a PR and 29 had a CR (48.4%). Eight patients (13.3%) had NC or PD. The objective response rate of all treated lesions was 86.6%. A total of 13 patients (21.7% overall, 44.8% of those with a CR) experienced a long-lasting response to ECT after one session and were free of disease after a mean follow-up of 27.5 months (Figure 1).

## Discussion

Malignant melanoma is the seventh most common type of cancer and the most common form of malignancy in young adults. The spread of malignant melanoma occurs by both lymphogenous and hematogenous routes. Local recurrence, in-transit metastases and satellitosis represent the same lymphatic dissemination process. The reported 5-year survival for patients with in-transit melanoma metastasis is poor, ranging from12% to 37% [4].

Treatment of recurrent cutaneous or subcutaneous tumors can be a challenge because of their unresectability and relative insensitivity to conventional systemic therapies. Recurrence represents a treatment concern for the physician and a source of distress and psychological burden for the patient, whose quality of life may be adversely affected by pain, ulceration, malodorous discharge and bleeding associated with lesions.

In general, the goal of treatment should be the elimination of local and systemic disease without undue toxicities or deformities, and with the consequent benefit of improved life expectancy and quality of life. ECT is a non-thermal tumor ablation modality, whereby the application of electric currents on cancer tissue renders the cell membrane permeable to non-or low permeant antineoplastic drugs, thus potentiating their cytotoxic effect directly inside the cellular DNA. This temporary permeability of the cell membrane caused by the electric pulses facilitates a potent localized effect and magnifies the drug cytotoxicity by several orders of magnitude [5-7]. Several cytotoxic drugs have been tested with the best candidate for this type of therapy appearing to be bleomycin. Neither electroporation nor bleomycin alone inhibits tumour growth, but the combination of both has a potent tumoricidal effect.

In preliminary reports, ECT with Bleomycin has been shown to be effective for the palliative treatment of metastatic cutaneous melanoma. In the ESOPE study, conducted by a consortium of four cancer centers in Europe, an objective response rate of 85% was achieved. A local tumor control rate of 88% was obtained for ECT with intravenous bleomycin, compared to 73% with intratumoral bleomycin and 75% with intratumoral cisplatinum. These results indicated that the intravenous route provides better results with minimal and tolerable side effects [8,9].

In a series of 14 refractory/relapsed patients with metastatic cutaneous disease, Quaglino et al. reported a response rate of 93% (13/14) after the first ECT, with a complete regression in seven patients (50%). Repeating the ECT procedure increased the response rate in the re-treated lesions (72%) with a local tumor control rate of 74.5% at 2 years [10]. Other reports also show the efficacy of ECT in the treatment of in-transit or subcutaneous metastases from cutaneous melanoma [11-13]. Recently, Moller et al. reported an objective response rate of approximately 80–90% in the palliative management of unresectable recurrent disease [14]. Kis et al. reported their experience with the use of ECT with bleomycin in 158 cutaneous and subcutaneous metastases from nine patients with cutaneous melanoma, in which an objective response rate of 62% was achieved [15]. Colombo et al. concluded that ECT is easy to perform and provides a good quality of life and economic benefits without the potentially undesirable side effects of systemic chemotherapy [16]. ECT has also been shown to rapidly stop bleeding in patients with bleeding cutaneous lesions, thereby improving quality of life [17].

A long-term complete response of cutaneous or subcutaneous metastases is believed to be difficult to obtain because of the intralymphatic spread of tumor cells. When surgical excision is not feasible due to the extension of the cutaneous disease, isolated limb perfusion could be an option, but its complexity and generally short duration of disease-free interval limits its indication [18]. Snoj et al. reported a long-lasting response in melanoma treated with ECT obtained with an easy, fast and effective procedure which can be repeated as much as need as shown by our study [19]. We found that approximately half of patients with a CR at 3 months (44.8%) maintained a long-lasting response (mean follow-up of over 2 years). This is the first study to report long-term response after ECT in patients with relapsed or refractory cutaneous melanoma.

## Conclusions

Treatment of early and multiple unresectable cutaneous recurrences of melanoma is a major therapeutic problem. The favorable outcome obtained in the present study shows that ECT is a reliable, easy, fast and effective procedure that provides benefits in terms of curative and palliative treatment for unresectable cutaneous lesions without adversely impacting on the quality of life of patients.

## Abbreviations

ECT: Electrochemotherapy; ESOPE: European standard operating procedures of Electrochemotherapy; WHO: World health organization; PD: Progressive disease; NC: No change; PR: Partial response; CR: Complete responses.

## Competing interests

All authors declare that there were no conflicts of interest in the preparation of this manuscript or in the design and conduct of the study.

## Authors’ contributions

CC have made substantial contributions to design the study and in drafting the manuscript. NM have conceived the study. UM, ES, LB, GDM, MLDC have been involved in acquisition, analysis and interpretation of data. GB, PAA have made general supervision of the manuscript. All authors read and approved the final manuscript.

## Pre-publication history

The pre-publication history for this paper can be accessed here:

http://www.biomedcentral.com/1471-2407/13/564/prepub
